# Cross-Regional View of Functional and Taxonomic Microbiota Composition in Obesity and Post-obesity Treatment Shows Country Specific Microbial Contribution

**DOI:** 10.3389/fmicb.2019.02346

**Published:** 2019-10-17

**Authors:** Daniel A. Medina, Tianlu Li, Pamela Thomson, Alejandro Artacho, Vicente Pérez-Brocal, Andrés Moya

**Affiliations:** ^1^Laboratorio de Biotecnología Aplicada, Facultad de Medicina Veterinaria, Universidad San Sebastián, Puerto Montt, Chile; ^2^Chromatin and Disease Group, Cancer Epigenetics and Biology Programme (PEBC), Bellvitge Biomedical Research Institute (IDIBELL), Barcelona, Spain; ^3^Epigenetics and Immune Disease Group, Josep Carreras Leukaemia Research Institute (IJC), Barcelona, Spain; ^4^Departamento de Ingeniería Química y Bioprocesos, Escuela de Ingeniería, Pontificia Universidad Católica de Chile, Santiago, Chile; ^5^Genomics and Health Area, Fundación para el Fomento de la Investigación Sanitaria y Biomédica de la Comunidad Valenciana (FISABIO)-Salud Pública, Valencia, Spain; ^6^Integrative Systems Biology Institute, University of Valencia, CSIC, Valencia, Spain; ^7^Biomedical Research Centre Network for Epidemiology and Public Health (CIBEResp), Madrid, Spain

**Keywords:** human gut microbiota, bariatric surgery, obesity, functional redundancy, metagenomic, functional convergence

## Abstract

Gut microbiota has been shown to have an important influence on host health. The microbial composition of the human gut microbiota is modulated by diet and other lifestyle habits and it has been reported that microbial diversity is altered in obese people. Obesity is a worldwide health problem that negatively impacts the quality of life. Currently, the widespread treatment for obesity is bariatric surgery. Interestingly, gut microbiota has been shown to be a relevant factor in effective weight loss after bariatric surgery. Since that the human gut microbiota of normal subjects differs between geographic regions, it is possible that rearrangements of the gut microbiota in dysbiosis context are also region-specific. To better understand how gut microbiota contribute to obesity, this study compared the composition of the human gut microbiota of obese and lean people from six different regions and showed that the microbiota compositions in the context of obesity were specific to each studied geographic location. Furthermore, we analyzed the functional patterns using shotgun DNA metagenomic sequencing and compared the results with other obesity-related metagenomic studies, we observed that microbial contribution to functional pathways were country-specific. Nevertheless, our study showed that although microbial composition of obese patients was country-specific, the overall metabolic functions appeared to be the same between countries, indicating that different microbiota components contribute to similar metabolic outcomes to yield functional redundancy. Furthermore, we studied the microbiota functional changes of obese patients after bariatric surgery, by shotgun metagenomics sequencing and observed that changes in functional pathways were specific to the type of obesity treatment. In all, our study provides new insights into the differences and similarities of obese gut microbiota in relation to geographic location and obesity treatments.

## Introduction

It has been established that the composition of human gut microbiota greatly influences human health (Carding et al., 2015, reviewed by Hall et al., 2017). Although the microbiota composition of each individual is relatively stable across adult life, it varies widely between individuals (Lozupone et al., 2012; Kolde et al., 2018). It has been observed that the human gut microbiota is primarily dominated by the phyla Firmicutes and Bacteroidetes (Zhernakova et al., 2016), with lesser contribution from Actinobacteria, Proteobacteria and Verrucomicrobia (Qin et al., 2010). Nevertheless, the microbiota composition differs geographically, primarily based on a variety of factors including host genetics, dietary habits, age, geographic location and lifestyle (Yatsunenko et al., 2012; Chilton, 2014; Nishijima et al., 2016; Fujio-Vejar et al., 2017; Gupta et al., 2017). To date, most of the studies on human gut microbiota have focused on populations from North America and Europe and although several of studies have demonstrated associations between microbiota alterations and diseases, including obesity, the specific contribution of these alterations to treatment response and how they differ across geographic locations still need to be envisioned.

Worldwide, obesity has nearly tripled since 1980 (Stevens et al., 2012). Information published by the World Health Organization (2016) shows that more than 1.9 billion adults, 18 years and older, have a body mass index (BMI) above 25 kg/m^2^ among which, over 650 million have a BMI > 30 kg/m^2^, hence classifying them as obese. Overweight and obesity are defined as abnormal or excessive fat accumulation due to environmental and genetic factors (Angelakis et al., 2012). While overweight individuals haves a BMI range between 25 and less than 30 kg/m^2^, obesity is classified by BMI as: obesity grade 1 (30 to 35 kg/m^2^), grade 2 (35 to 40 kg/m^2^) and grade 3 (40 to 60 kg/m^2^) (NHLBI Expert Panel, 1998). Diverse studies have associated obesity with altered gut microbiota and reduced functional potential (Ley et al., 2006; Turnbaugh et al., 2006; Tremaroli and Backhed, 2012; Karlsson et al., 2013; Le Chatelier et al., 2013; Damms-Machado et al., 2015; Haro et al., 2016; Medina et al., 2017), which can be partially reversed after surgical intervention (Palleja et al., 2016). It has been originally observed that the relative abundance of Firmicutes and Bacteroidetes can be altered in obese patients, where an over-representation of Firmicutes is observed, in contrast to their lean counterparts (Ley et al., 2006; Turnbaugh et al., 2009; Million et al., 2012; Walters et al., 2014; Kasai et al., 2015; Haro et al., 2016). These taxonomic differences between lean and obese subjects may contribute to the development and perpetuation of obesity in several ways, including fat storage, regulation of energy metabolism, energy extraction from short chain fatty acids, increased low-grade inflammation and altered bile acid metabolism (Qin et al., 2012; Karlsson et al., 2013; Khan et al., 2016). However, a recent study focusing on the meta-analysis of previously published data using forest machine learning models showed no significant differences in gut microbiota composition between obese and healthy individuals (Sze and Schloss, 2016). Nevertheless, further research is required to unravel the exact role of gut microbiota composition in obesity context, and the factors, including geographical location, that may have an influence on not only differential microbial abundance but also long-term patient outcome.

Gastric surgical procedures, commonly known as bariatric surgery, have been successful in mediating long-term weight loss and reducing the incidence of related comorbidities (Sjöström, 2008; Eldar et al., 2011). Roux-en-Y gastric bypass (RYGB) is one of the most common bariatric surgery procedures in the United States (Smoot et al., 2006), where a small stomach pouch is connected to the proximal jejunum to directly bypass food to the small intestine, resulting in restrictive and malabsorptive nutrient intake (Tice et al., 2008; Tran et al., 2016). Another common bariatric surgery is sleeve gastrectomy (SG), where a significant portion of the stomach is removed to decrease its volume, leading to a significant reduction in the amount of food consumed (Gumbs et al., 2007). Since their invention, a number of studies have observed changes in obesity-associated microbiota and functional gene richness following different types of bariatric surgery (Zhang et al., 2009; Graessler et al., 2013; Kong et al., 2013; Tremaroli et al., 2015; Shao et al., 2016; Ilhan et al., 2017; Medina et al., 2017; Murphy et al., 2017). Specific changes following surgical intervention include an increase in Proteobacteria (*Escherichia coli*, *Enterobacter* spp.), changes in *Bacteroides* and *Prevotella* abundance, accompanied by an increase in *Akkermansia* and a decline in *Clostridium* genus, and global changes in Firmicutes/Bacteroidetes phyla ratios (Zhang et al., 2009; Furet et al., 2010; Li et al., 2011; Kong et al., 2013; Damms-Machado et al., 2015; Louis et al., 2016; Palleja et al., 2016; Ilhan et al., 2017; Medina et al., 2017). These microbial changes following bariatric surgery have shown to mediate different outcomes and, interestingly, such changes appear to depend on the type of surgery performed (Ilhan et al., 2017; Medina et al., 2017; Murphy et al., 2017). Specifically, metagenomic studies following RYGB showed an increase in pathways involving aerobic respiration and glutathione transfer and metabolism (Palleja et al., 2016). Both observations were in agreement with the increase in Proteobacteria phylum abundance, driven by facultative anaerobes, such as *E. coli*, as a result of lower gastric acid exposure during stomach transit. In the same study, the authors found an increase in the pathways that degrade putrescine to succinate to produce gamma-aminobutyric acid (GABA) as a byproduct. GABA is known to act on receptors in the hypothalamus to promote satiety and, additionally, is thought to promote GLP-1 release, a positive regulator of GABA production by pancreatic beta-cells, which provides further involvement of this pathway following RYGB (Palleja et al., 2016). In addition, a complementary study conducted 9 years after RYGB intervention showed that bacterial rearrangements were stable in the long run and surgically altered microbiota promoted reduced fat deposition in recipient mice (Tremaroli et al., 2015).

In this study, we compared the gut microbiota composition and functional patterns of obese subjects from Chile with published data from Italy, Denmark, United States, France, and Saudi Arabia, in order to better understand the microbiota contribution to obesity. We found obese subjects to show geographic specificity in regards to relative microbiota abundance, similar to what has been previously observed in healthy individuals. Interestingly, the gut microbiota of obese patients did not display differential enrichment of functional pathways between countries, indicating that the geography-specific microbial compositions converge to perform similar functions in obese individuals. Furthermore, this study analyzed the metagenomic profiles of Chilean patients subjected to gastric bypass and sleeve gastrectomy and observed changes in the functional capacity of gut microbiota after surgery. Specifically, we identified *Akkermansia muciniphila* as one of the bacteria that drives the change in metabolic pathways after surgical intervention for obesity in Chilean patients.

## Materials and Methods

### Sample and DNA Raw Data Collection

DNA raw data sequences from the stool of obese and lean subjects were obtained from studies carried out in Chile (Medina et al., 2017; Thomson et al., 2019), Italy (Tremaroli et al., 2015), Denmark (Palleja et al., 2016), United States (Ilhan et al., 2017), France and Saudi Arabia (Yasir et al., 2015), described in Table 1 and Supplementary Table S1. In addition, 12 DNA stool samples from Chilean obese patients before and after treatment obtained from a previous study (Medina et al., 2017) were sequenced using shotgun metagenomics. All experiments were conducted in accordance with the Declaration of Helsinki and approved by the Ethics Committee of the Faculty of Medicine, Pontifical Catholic University of Chile (n° 15-337).

**TABLE 1:** Description of studies used in this work.

**Study**	**Country**	**Methodology**	**N° lean subjects**	**N° obese subjects**	**DOI**
Medina et al., 2017	Chile	16S rDNA Amplicon	0	19	doi: 10.7717/peerj.3443
Thomson et al., 2019	Chile	16S rDNA Amplicon	28	0	doi: 10.1017/S0007114519001570
Yasir et al., 2015	France	16S rDNA Amplicon	12	16	doi: 10.1038/nutd.2015.3
Yasir et al., 2015	Saudi Arabia	16S rDNA Amplicon	9	9	doi: 10.1038/ismej.2017.71
Ilhan et al., 2017	United States	16S rDNA Amplicon	10	15	doi: 10.1038/ismej.2017.71
Medina, this study	Chile	Shotgun metagenomics	0	6	This study
Palleja et al., 2016	Denmark	Shotgun metagenomics	0	13	doi: 10.1186/s13073-016-0312-1
Tremaroli et al., 2015	Italy	Shotgun metagenomics	0	7	doi: 10.1016/j.cmet.2015.07.009

### Taxonomic Profiling From 16S rDNA Gene Amplicon Sequencing

Raw data of DNA sequences belonging to different studies were downloaded from ENA-EMBL or SRA-NCBI databases (Supplementary Table S1). To access the microbiota taxa and abundance, in this study we re-analyzed all raw data sequences using Microbiome Helper v2.3 OVA pipeline (Comeau et al., 2017). Briefly, MiSeq paired-end sequences were joined using PEAR (Zhang et al., 2014). Joined sequences were filtered by quality and length, in which demultiplexing and barcode depletion were performed using Microbiome Helper and QIIME v1.9.1 scripts (Caporaso et al., 2010; Navas-Molina et al., 2013; Comeau et al., 2017). Chimera sequences were filtered using the Vsearch tool (Rognes et al., 2016). Operational taxonomic units (OTUs) were picked by open-reference command and defined by clustering at 3% divergence (97% similarity) using the GreenGenes database release 08-2013 as reference (DeSantis et al., 2006; McDonald et al., 2012). Diversity analyses were performed using QIIME v1.9.1 scripts under Microbiome Helper v2.3 environment. The sequencing depth for even sub-sampling and maximum rarefaction depth was at least 10000 counts/sample, regard the minimum value obtained after OTU-picking for each data set (Supplementary Table S2). Predictive metagenomic functional profiling of microbial composition were performed using PICRUSt (Langille et al., 2013), following the instructions provided in their Metagenomic Prediction Tutorial. KEGG Orthology analyses were performed using the “ko_to_pathway_map” PICRUSt database to identify the enrichment of metabolic pathways.

Taxonomic abundance and metagenomic prediction tables were exported to R environment (R Core Team, 2013) for statistical analysis and figures were represented using the package LSD Lots of Superior Depictions (Schwalb et al., 2011). Volcano plots were constructed using the adjusted FDR *p*-value obtained from the unpaired *t*-test comparisons and the fold change of each condition. The taxonomic processed data obtained from QIIME and the metadata for the raw data files used in this study are exhibited in Supplementary Table S2.

The PICRUSt functional prediction was validated comparing the KEGG Orthology abundances obtained with the Uniref90 metagenomic abundances from the HUMAnN2 output. For this, the metagenomic Uniref90 table was converted to KEGG Orthology using the script humann2_regroup_table from HUMAnN2 (Abubucker et al., 2012). Both KEGG Orthology datasets were merged by row and normalized using quantile normalization to compare linear dependence between each data set using Pearson and pairwise Spearman rank correlation in the R environment (R Core Team, 2013).

### Functional Annotation and Metagenomic Profiling of Fecal DNA

A total of 12 DNA stool samples were sequenced using the Illumina HiSeq next-generation sequencing platform carried out at Genoma Mayor (Universidad Mayor, Chile) with an output of 2 × 100 pb and 20 × 10^6^ paired-end reads per sample. The raw data produced from DNA sequencing in this study were stored at the ENA-EMBL database under the accession number PRJEB29060. To evaluate the taxa abundance and metagenomic composition, all metagenomic raw sequences were analyzed in parallel using the Microbiome Helper v2.3 environment following the metagenomics SOP v2 tutorial. This pipeline allowed the calculation of microbiota abundance using functions from MetaPhlAn2 (Truong et al., 2015) and functional profile using HUMAnN2 (Abubucker et al., 2012). DNA sequence quality control was performed using KneadData (McIver et al., 2018), in which low quality sequences were first removed by Trimmomatic (Bolger et al., 2014), followed by the application of Bowtie2 to screen out the contaminant DNA sequences mainly from human and viruses (Langmead and Salzberg, 2012). Both programs were run simultaneously for all samples using the GNU Parallel tool to repeat concatenate the entire process for each data set (First and Job, 2011). The paired-end files were merged using the script provided by the Microbiome Helper pipeline and then taxonomic and functional profiling were performed using MetaPhlAn2 and HUMAnN2, respectively. Gene family and pathway abundances were normalized for each sample and represented as a percentage. The resulting tables were exported to the R environment (R Core Team, 2013) for statistical analysis and figure representation. Metadata sets used for DNA shotgun sequencing to obtain stratified and unstratified metagenomic profiles are listed in Supplementary Table S3.

### Data Comparison and Statistical Analysis

Statistical analyses were conducted using the R environment (R Core Team, 2013) version 3.4.4 (2018-03-15). Before calculations between countries, data sets were scaled proportionally, and using the R package preprocessCore (Bolstad, 2019), quantile normalization was performed to reduce batch effects between data belonging to different sources as was previously used for genome-wide analyses (Sun et al., 2011; Guo et al., 2014; Fei et al., 2018). For differential analysis of group variance between lean and obese microbiota abundance, we used Canonical Correspondence Analysis (CCA, a.k.a. constrained correspondence analysis) and Adonis tests, in which both were calculated using the R package vegan (Oksanen et al., 2018). The Adonis test was used for permutational multivariate analysis of variance using distance matrices, in which samples were fitted to linear models to calculate the whole compositional variability taking into account different sources of variation as well as the interactions between them. Spearman’s rank correlation analyses were used to assess taxa abundance or functional abundance associations between the samples groups. To analyze differences in group means between lean and obese microbiota abundance, we applied Wilcoxon sum rank test with FDR adjustment utilizing the Benjamini Hochberg (BH) method. This was performed using the R command p.adjust(), in which we considered tests with FDR < 0.05 to be significant. In order to identify differentially enriched biomarkers among the compared groups, we applied the LEfSe analytic method using the online interface Galaxy^1^ (Segata et al., 2011). Statistical differences between the KEGG Orthology abundances were calculated using the unpaired *t*-test and adjusted by BH FDR as described above. –log_10_ FDR was plotted against log_2_ ratio of each country in respect to Chile and represented using the R package LSD Lots of Superior Depictions (Schwalb et al., 2011).

## Results

### Gut Microbial Diversity Is Specific to Geographic Locations

**FIGURE 1:**
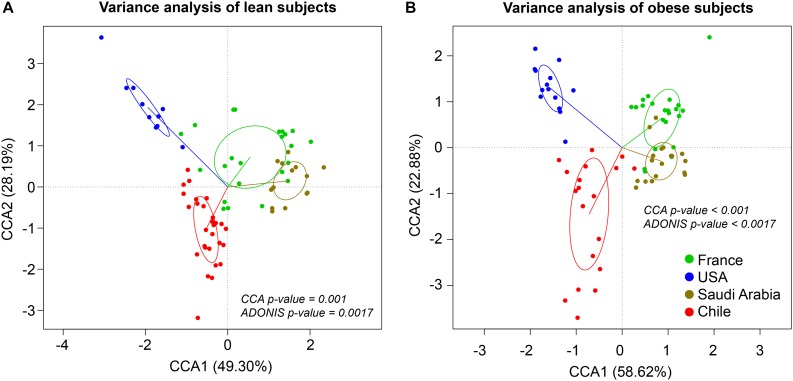
Taxonomic abundance comparison at genus level from 16S rDNA sequencing. Constrained Correspondence Analysis (CCA) and Adonis test were performed to assess the variance in microbiota profiles at the genus level in lean **(A)** and obese **(B)** subjects. Vectors represent quantitative explanatory variables with confidence circles depicted for each country. Corresponding *p*-values are shown for each analysis and *p*-value < 0.05 was considered statistically significant. *x*- and *y*-axis show CCA1 and CCA2 components, respectively. Green, blue, brown and red points indicate individuals from France, United States, Saudi Arabia, and Chile, respectively.

**FIGURE 2:**
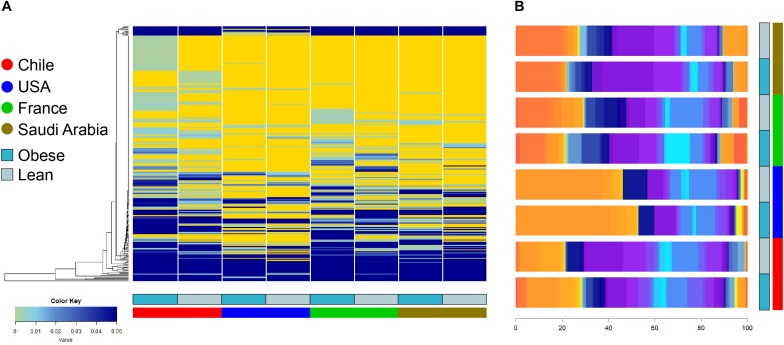
Heatmap clustering and stacked bar plot of taxonomic abundances of lean and obese subjects at genus level. **(A)** Taxonomy abundance at genus level between obese and lean subjects were clustered using complete linkage method. Values indicate abundance of microbiota at the genus level. **(B)** Average taxonomy abundance at genus level were represented as stacked bar plot to of lean and obese subject of each country. *x*-axis depicts percentage of abundance of each taxonomy. Dark and light blue represent obese and lean subjects, respectively. Red, blue, green, and brown represent individuals from Chile, United States, France, and Saudi Arabia, respectively.

The composition and function of the human gut microbiota represent one of most important factors involved in obesity and its treatment (Ley et al., 2006; Tremaroli and Backhed, 2012; Karlsson et al., 2013; Le Chatelier et al., 2013; Damms-Machado et al., 2015; Medina et al., 2017). It has been previously established that the human gut microbiota displays pronounced differences between individuals residing in distinct geographic locations (Yatsunenko et al., 2012; Fujio-Vejar et al., 2017; Gupta et al., 2017). It is therefore of great interest to compare the microbial diversity of obese individuals from different geographical locations around the world. In this line of research, this study compared the gut microbial diversity of obese and lean subjects from Chile (Medina et al., 2017; Thomson et al., 2019) with data published by other studies in different regions around the world, namely United States (Ilhan et al., 2017), France and Saudi Arabia (Yasir et al., 2015), described in Table 1. Taxonomic microbiota abundance were collected by sequencing the 16S rDNA hypervariable regions V3–V4 or V4–V5 using the Illumina MiSeq platform (Supplementary Table S1), and raw data was processed as described in materials and methods. Taxonomic abundance comparisons at the genus level from the data obtained showed significant diversity in microbiota composition in lean people as previously reported (Yatsunenko et al., 2012; Nishijima et al., 2016; Fujio-Vejar et al., 2017; Gupta et al., 2017), but also is country specific in gut microbiota of obese subjects. Specifically, data variance analysis using CCA and Adonis tests showed significant differences (*p*-value < 0.05) in the gut microbiota composition between Chile, United States, France, and Saudi Arabia, in which the lean and obese microbiota were clustered by country (Figures 1A,B). Utilizing a different approach, pairwise Spearman rank correlation, we further demonstrated that the taxonomic abundance correlation of obese gut microbiota differed between countries (Supplementary Figure S1A). Nevertheless, although obese microbiota composition is clustered by country, there is significant inter-individual variability between subjects, indicated by high dispersion patterns observed in data variance analyses (Figure 1). In this regard, although some proximity is observed between France and Saudi Arabia microbiota, CCA and Adonis test variance analyses between the two countries shows a significant statistical difference (Supplementary Figure S1B). In all, these comparisons indicate that the microbiota compositions of obese patients and lean subjects were specific to each analyzed geographic location and displayed significant differences between countries. Comparing taxonomic abundance of obese individuals with their lean counterparts from the same country we observed significant differences by multivariate variance analyses for Chile, France, and Saudi Arabia (CCA *p*-value < 0.05); however, no statistically significant differences were observed for United States (Supplementary Figure S2). Wilcoxon sum-rank tests showed significant differences (FDR < 0.05) between lean and obese microbiota in Chile and France data sets, but not in United States and Saudi Arabia. Interestingly, although CCA showed clustering by country in both cases, France and Chile shared some genus changes between lean and obese microbiota (*Megasphaera*, *Veillonella*, *Adlercreutzia* and *Lachnospira*) (Supplementary Table S2). In addition, heatmap clustering by complete linkage method and the stacked bar plot of taxonomic abundances showed differential patterns not only between obese and lean subjects, but also between countries, which again highlights our observation that taxonomic microbial distribution is specific to each country (Figure 2).

**FIGURE 3:**
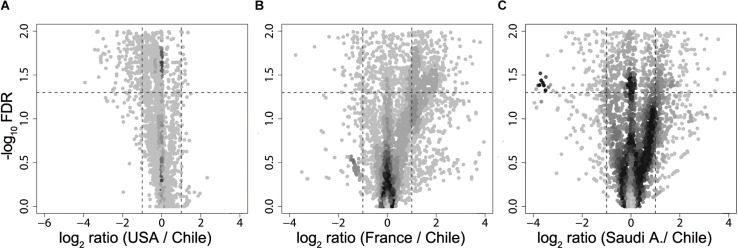
Volcano plot of KEGG Orthology abundances obtained from metagenomic simulation. Fold change of United States **(A)**, France **(B)**, and Saudi Arabia **(C)** of KO abundance with regard to Chile are plotted against –log_10_ of FDR. Dotted lines in the *x*-axis denotes a two-fold change, while dotted lines in the *y*-axis delimit a FDR value of 0.05.

Next, using the PICRUSt tool (Langille et al., 2013) designed to infer the functional patterns from taxonomic profile, we compared predicted KEGG Orthology (KO) enrichment of obese subjects from United States, France, and Saudi Arabia with the KO obtained from the obese Chilean data as reference in order to identify potential functional differences. Here, we observed significant differences (FDR < 0.05) in enriched pathways for all the countries studied with respect to Chile (Figure 3). Downregulated pathways in Saudi Arabia displayed an enrichment in translation and energy metabolism, whereas the United States and France showed a downregulation in membrane transport. For upregulated pathways, United States showed a specific enrichment in cellular processing and signaling and amino acid metabolism, whereas France and Saudi Arabia were found to enrich in pathways involving membrane transport (Supplementary Figure S3). In summary, the differences observed by functional prediction suggested that the human gut metagenome of obese subjects, like their healthy counterparts (Yatsunenko et al., 2012), may differ according to the geographical location.

### Metagenomic Variations Across Regional Location in the Context of Obesity

**FIGURE 4:**
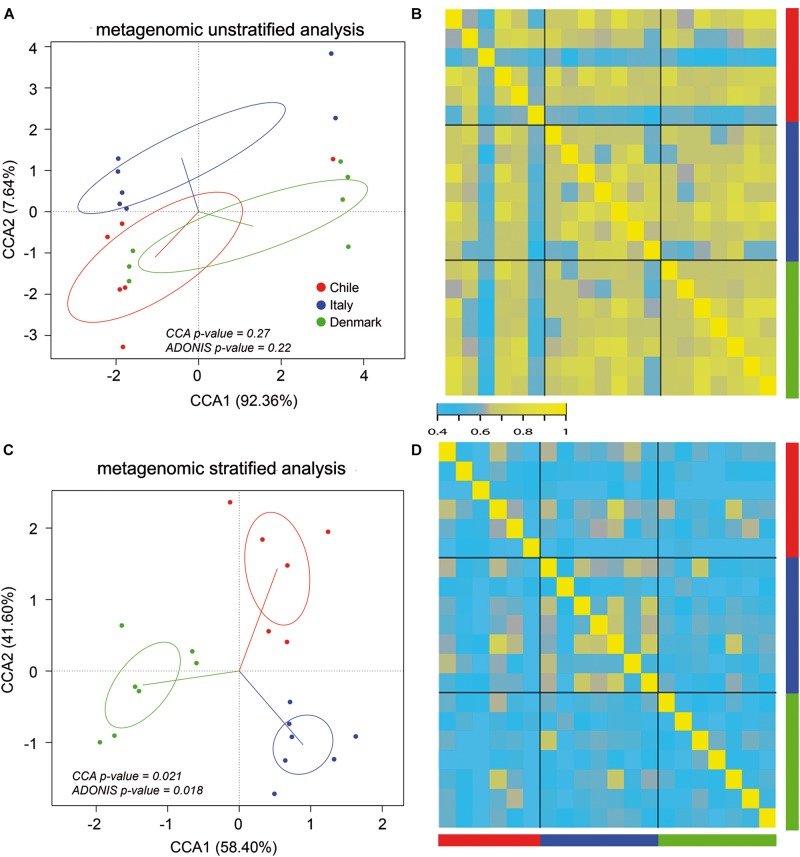
Stratified and unstratified functional profiles of obese gut microbiota from three different countries. Unstratified **(A,B)** and stratified **(C,D)** analyses of obese gut microbiota abundance for Chile (red), Italy (blue) and Denmark (green). Scatterplots **(A,C)** represent CCA and Adonis test analysis, and heatmap **(B,D)** represent pairwise Spearman rank correlation of functional microbial abundance between countries.

**FIGURE 5:**
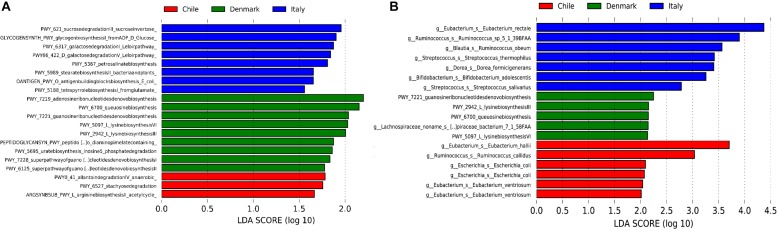
Linear discriminant analysis (LDA) effect size (LEfSe) of metabolic pathways. Unstratified **(A)** and stratified **(B)** functional enrichment analyses from microbiome of obese subjects belonging to Chile (red, *n* = 6), Denmark (green, *n* = 7) and Italy (blue, *n* = 7). Pathways with LDA scores higher than 1.5 **(A)** or 2 **(B)** are shown. For stratified functional enrichment **(B)**, unmapped/unintegrated reads are denoted as the bacterial taxa in which the pathways belong to.

Our initial analysis utilizing 16S rDNA amplicon sequencing showed marked differences between the compositions of the gut microbiota of obese subjects from different countries, therefore, to further validate our initial observation, we analyzed stool samples of 6 Chilean pre-treatment obese patients obtained from a previous study (Medina et al., 2017) by shotgun DNA sequencing in order to dissect the contribution of metabolic functional changes and microbiota compositions. Additionally, we compared the results obtained from the Chilean cohort with similar studies carried out from patients in Italy (Tremaroli et al., 2015) and Denmark (Palleja et al., 2016), described in Table 1. All raw data were analyzed using the Metagenomic SOAP v2 from Microbiome Helper (Comeau et al., 2017) as described in “Materials and Methods.” First, we compared the functional composition of obese subjects from Chile (*n* = 6), Italy (*n* = 7) and Denmark (*n* = 7) before any medical intervention utilizing CCA, Adonis tests and pairwise Spearman rank correlation. Initially, we performed an unstratified analysis, which corresponds to the analysis of metabolic pathway enrichment without taking into account microbial composition, and observed no statistically significant differences between the three countries using CCA analysis and Adonis tests (*p*-value > 0.05), in which the variance plot displayed little dispersion between the samples from different countries (Figure 4A). Furthermore, the pairwise Spearman rank correlation showed overall good correlation between the samples of all datasets (Figure 4B). Hence, in contrast to PICRUSt prediction, these results showed that the overall microbial functionality had no differences between obese subjects belonging to different world regions. Subsequently, we performed a stratified data analysis, a type of analysis that distinguishes the functional contribution of each bacterial species to the overall metabolic pathways. Interestingly, stratified data, unlike unstratified analysis, showed significant dispersion between the countries (Figure 4C), and no correlation between datasets was observed (Figure 4D), indicating that individual microbial metagenomic contribution to overall functional pathways is different between countries, in agreement with functional inferences obtained by PICRUSt (Figure 3). Also, we use the Spearman rank correlation values obtained to compare statistically into-countries and cross-country associations in both unstratified and stratified data, finding no statistical differences between Spearman values (*p*-value FDR adjusted of 0.75 and 1, respectively). Utilizing linear discriminant analysis (LDA) to quantify phylogenetic and functional diversity, pathways over 1.5 LDA score were found to be differentially enriched between countries in both stratified and unstratified approaches (Figure 5). In addition, we validated the PICRUSt functional prediction comparing KEGG Orthology obtained with HUMAnN2 output from metagenomic data using Pearson and pairwise Spearman rank Correlation, with both showing linear dependence between 3590 common KEGG Orthology families (Supplementary Figure S4 and Supplementary Table S5), which proved the relationship between *in silico* predictions and our biological findings as was previously demonstrated by PICRUSt authors (Langille et al., 2013). Altogether, these results suggested that microbial contribution to functional pathways was country-specific, indicating that there was redundancy in the functions of these distinct microbial species found in obese subjects from different countries, in which different microbiota components contribute to obtain similar metabolic outcomes.

Furthermore, microbial taxonomy abundance data obtained from shotgun DNA sequencing showed that Chilean microbiota composition from obese subjects was different compared to Italian and Danish cohorts (Tremaroli et al., 2015; Palleja et al., 2016). CCA analysis and Adonis tests showed significant differences between the microbiota of analyzed subjects at species level (Supplementary Figure S5A), which strengthens our previous observations obtained from the 16S rDNA sequencing taxonomic comparison at genus level (Figure 1B). In addition, LDA analysis showed differences in species abundance between the three microbial datasets species with and LDA scores higher than 2, reinforcing the observation that the microbiota under dysbiosis has different compositions specific to each geographical location (Supplementary Figure S5B).

### Gut Microbiota Functionality Changes Following Bariatric Surgery in Chilean Subjects

**FIGURE 6:**
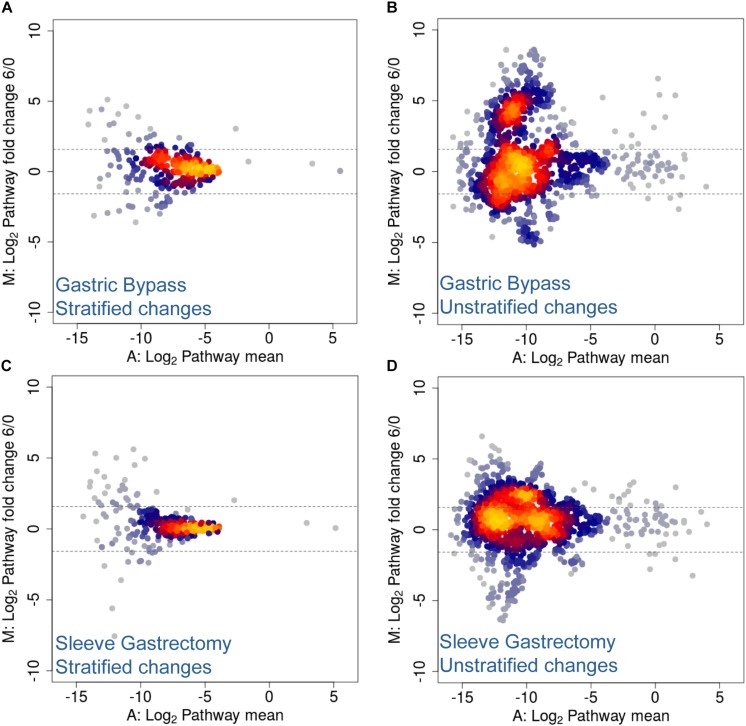
MA Plots for stratified and unstratified functional changes after bariatric surgery in Chilean patients. **(A,B)** Gastric bypass and **(C,D)** sleeve gastrectomy stratified **(A,C)** and unstratified **(B,D)** functional changes 6 months post-surgery. Values are expressed as mean (*x*-axis) and fold change (*y*-axis), and represented as log_2_ values. Dotted lines denote a two-fold change.

Several studies have shown that the regulation of energy and fat storage is influenced by intestinal microbes, and this composition may contribute to obesity (Qin et al., 2012; Karlsson et al., 2013). In this regard, it has been reported that human gut microbiota composition varies between obese patients who underwent different types of surgical intervention (Zhang et al., 2009; Furet et al., 2010; Schloss, 2016; Ilhan et al., 2017; Medina et al., 2017; Murphy et al., 2017). Moreover, several of these studies describe functional changes after treatment and its prevalence across time (Tremaroli et al., 2015; Palleja et al., 2016). More specifically, they report an increase in Proteobacteria abundance and facultative anaerobes, such as *E. coli*, and an enrichment in microbial pathways involving aerobic respiration, glutathione and gamma-aminobutyric acid metabolism. Similarly, in a previous study from our laboratory using Chilean obese patients, we observed significant changes in the human gut microbiota, and these changes were specific to the type of surgery performed (Medina et al., 2017). Here, utilizing the same cohort, we performed shotgun DNA sequencing of stool samples before and after surgical intervention in order to analyze changes in taxonomic composition (Medina et al., 2017). Although no statistical testing was performed due to insufficient cohort number (*n* = 2 for each group), our results suggested that the two kinds of bariatric surgery mediated different rearrangements of functional pathways. We found stratified and unstratified functional changes of several folds, 6 months following either Roux-en-Y gastric bypass (RYGB) or sleeve gastrectomy (SG) interventions (Figure 6). More specifically, unstratified functional changes were more pronounced in RYGB–treated patients compared to SG-treated patients, represented by higher data variance with an increase in log_2_ fold change (Figures 6B,D). Functional changes in unstratified RYGB data suggested an increase in pathways related with acetyl-CoA biosynthesis, trehalose degradation, GABA shunt and phospholipid remodeling. Contrastingly, changes observed in SG intervention included an increase in metabolic pathways such as fatty acid β-oxidation, TCA cycle and acetyl-CoA biosynthesis (Supplementary Table S4). Top stratified functional changes after RYGB were mainly driven by *A. muciniphila*, *E. coli*, *Bacteroides vulgatus*, *Eubacterium siraeum* and *Streptococcus salivarius*, while in SG functional changes were driven by *Bacteroides cellulosilyticus*, *S. salivarius*, *Eubacterium eligens*, *Lactococcus lactis*, *Alistipes finegoldii, E. coli* and *A. muciniphila* (Supplementary Table S4). Altogether, these results suggested that bariatric surgery in Chilean patients also caused functional rearrangement in microbiota, in concordance to previous metagenomic studies (Graessler et al., 2013; Tremaroli et al., 2015; Palleja et al., 2016), and these changes appeared to be specific to the surgery performed.

## Discussion

Obesity is a world-wide health problem whose global prevalence has increased at an accelerated rate since 1980 (Stevens et al., 2012). It has been previously described that the composition and functionality of human gut microbiota plays a crucial role in mediating both disease and recovery following medical intervention (Turnbaugh et al., 2006; Tremaroli and Backhed, 2012; Karlsson et al., 2013; Le Chatelier et al., 2013; Damms-Machado et al., 2015). This study compared the gut microbiota compositions of obese subjects from Chile with previously published data from the United States, France, and Saudi Arabia using 16S rDNA Illuminia MiSeq, as well as from Italy and Denmark using Illumina HiSeq shotgun metagenomics, and found that the microbiota composition differs significantly between obese and lean subjects from these different countries, showing country-specific clustering. It has previously been described that a number of factors can modulate the composition of intestinal microbiota, one of which is geographic origin (reviewed by Rojo et al., 2017). A previous study compared the microbiota composition of a group of healthy subjects from France with Saudi Arabia by 16S rDNA sequencing and suggested that the dietary habits of different regions or countries can generate changes in intestinal microbiota (Yasir et al., 2015). This idea was reinforced by other studies suggesting that different types of diets directly change microbiota composition (Wu et al., 2011; Fava et al., 2013). In fact, mice subjected to obesogenic diets displayed significant functional and taxonomic modifications in their gut microbiota (Tran et al., 2019). Other studies show that genetics play a secondary role compared to environmental factors (Gupta et al., 2017; Jones et al., 2018; Rothschild et al., 2018). However, twin studies revealed more highly correlated microbiota compositions between monozygotic twins than dizygotic twins (Goodrich et al., 2014). Given subjects belonging to close location have less genetic variance than subjects from distant regions, this may also contribute to the differences in microbiota composition observed between geographical origins.

Our work attempted to indicate the need for the global study of human microbiota for a proper assessment of the microbial contribution in respect to geographic distribution. In this context, we compared the taxonomic gut microbiota abundance of lean and obese at genus level using multivariate variance analysis and non-parametric mean comparison. Although, using Wilcoxon rank test, we found different statistical changes in France and Chile gut microbiota, this was, however, not the case for United States and Saudi Arabia. Our results suggest a country-specific signature for both lean and obese microbiota. Nevertheless, we cannot discard the possibility that our observations may be influenced by technical and methodological factors. First, it is known that non-parametric tests have less chance of detecting a true effect where one exists (Whitley and Ball, 2002). Hence, utilizing a different statistical approach to analyze differences in variance (CAA and Adonis test) between lean and obese subjects in France, Chile, and Saudi Arabia, the subsequent results support the notion that obese versus lean microbiota is different between countries. Second, we cannot discard that this observation may be relatively influenced by the methodology differences between studies and also may be confounded by large interpersonal variation. A meta-analysis of the association between differences in the microbiome and obesity status made between 10 published studies found that is difficult to classify a subject as obese by its microbiota composition, suggesting the possibility that each individual has their own bacterial signatures (Sze and Schloss, 2016). Another meta-analysis that compared lean and obese subject from five published studies also found that signatures of obesity were not consistent between studies, as just one of the 5 studies analyzed showed diversity differences between lean and obese subject (Walters et al., 2014). Nevertheless, the authors declared that it was possible to assess differences between lean and obese subjects when they changed the method to identify OTUs (from “close reference” to “*pick de novo*”) or used supervised learning tools to categorize subjects according to lean and obese states with considerable accuracy (Walters et al., 2014). Altogether, these observations suggest that geographical differences between lean and obese subjects requires additional validation using an international cohort that not only takes into account confounding factors but maintain the same experimental design.

Our study performed a functional inference analysis based on the data obtained by 16S rDNA sequencing and observed significant differences in KEGG Orthology enrichment in samples obtained from France, United States, and Saudi Arabia compared to Chile. These results suggested the microbial differences observed were associated with changes in functional pathways, however, this kind of analysis was unable to differentiate the microbial contribution of each microorganism species to the identified functional pathways. Therefore, a more detailed comparison of metagenomic DNA sequencing data was used, which was obtained using the HiSeq platform to analyze stool samples of obese patients from Chile, Italy and Denmark. First, we performed an unstratified analysis, a type of metabolic pathway enrichment analysis without taking into account microbial contribution, and revealed that the functionality in obesity context did not show statistical differences (in both CCA and Adonis testing) between patients from Chile, Italy and Denmark, suggesting that gut microbiota metabolic pathways do not change according to geographic origin. Conversely, using a stratified approach that could identify specific microbial composition and their individual contribution to metabolic pathways, significant differences between countries were identified, in agreement with our initial PICRUSt metabolic inference, because both methods takes in account taxonomic composition to assess metabolic pathways abundance. Altogether, our observations indicate while overall metabolic functions do not change, microbial functional contribution to obesity are specific to each country.

Although the lack of differences observed in metagenomic unstratified analysis may be due to small cohort numbers, our comparisons suggest that the gut microbiota of obese patients from different countries have functional convergence, where the same essential metabolic functions are carried out by related and unrelated bacterial species. Nevertheless, it is not surprising that bacterial compositions of obese individuals differ between counties due to both genetic and environmental factors, as previously described by others (reviewed by Gupta et al., 2017), however, our observations relating to convergent metabolic functions provide new insights into how the gut microbiota shape common disease phenotypes. The concept of intestinal functional redundancy has been previously explored, in which taxonomic diversity appears to be irrelevant for the inference of functional traits (reviewed by Moya and Ferrer, 2016; Rojo et al., 2017). One such example was demonstrated by the Human Microbiome Project Consortium (Huttenhower et al., 2012), where the authors observed the microbial metabolism to remain constant across individuals over time despite high variability in composition. One possible explanation of microbial functional redundancy can be due to the evolutionary convergence of unrelated taxa, in which variable combinations of species from different phyla can at least partially fulfill the metabolic functions of another, resulting in different species of bacteria behaving similarly (Moya and Ferrer, 2016; Rojo et al., 2017).

Bariatric surgeries have become a frequent choice of treatment for obesity patients over the last years, being SG the most frequent procedure in the world (Angrisani et al., 2017; reviewed by ASMBS, 2018). Several studies have shown that the intestinal microbiota composition changes following surgical intervention and these changes vary between patients who have undergone different kind of surgical intervention such as RYGB, SG and laparoscopic adjustable gastric banding (LAGB) (Tremaroli et al., 2015; Palleja et al., 2016; Ilhan et al., 2017; Medina et al., 2017). One of the limitations of our metagenomic study after bariatric surgery is our small cohort number (*n* = 2 for each group), and therefore, further studies with larger cohort are required to confirm that different treatments mediate differentially metagenomic rearrangements. However, this study suggest that the microbial functionality of patients changed 6 months following surgical treatment. In agreement with previous studies, it also observed specificity in the changes regarding to the type of surgery performed, where functional changes were mainly mediated by *A. muciniphila*, *E. coli*, *B. vulgatus*, *E. siraeum* and *S. salivarius* after RYGB, and by *B. cellulosilyticus*, *S. salivarius*, *E. eligens*, *L. lactis*, *A. finegoldii*, *E. coli* and *A. muciniphila* species after SG. Although it is impossible to determine from this study whether the microbiota composition changes were a consequence of dietary changes or weight modification, our results not only hint at a microbiota signature for different bariatric surgeries, but also suggest the need for further microbiota meta-analyses at world-wide levels to study metabolic disorders such as obesity.

A common key factor in obesity is the low abundance of *A. muciniphila* (Schneeberger et al., 2015; Palleja et al., 2016; Medina et al., 2017; Seck et al., 2018), a mucin-degrading bacterium that resides in the human gut mucus layer and whose abundance in healthy subjects represents 3–5% of the residential microbial community (Derrien et al., 2004). Interestingly, it has been shown that *A. muciniphila* prevents inflammation and adipose tissue alterations in mice (Schneeberger et al., 2015). The administration of *A. muciniphila* grown under mucin-depleted conditions is effective in reducing obesity and improve intestinal barrier integrity in obese mice (Shin et al., 2019), controls fat mass storage and glucose homeostasis in obese and type 2 diabetic mice (Everard et al., 2013), and it has been previously described that bariatric surgery improves its abundance (Damms-Machado et al., 2015; Tremaroli et al., 2015; Palleja et al., 2016; Medina et al., 2017). Furthermore, overweight and obese individuals with higher *A. muciniphila* abundance is associated with a healthier metabolic status compared with lower abundance (Dao et al., 2016). Therefore, the enrichment of *A. muciniphila* provides a possible therapy in the treatment of obesity, and this possibility has been explored in a recent study (Sheng et al., 2018). Here, we identified *A. muciniphila* as one of the bacteria that drove the changes in metabolic pathways after surgical intervention of Chilean obese patients, an important observation considering that gut microbiota of healthy Chilean subjects has high abundance of *Verrucomicrobia* bacteria, including *A. muciniphila* (Fujio-Vejar et al., 2017). Further studies to understand the underlying mechanisms involving *A. muciniphila* as a target of bacto-therapy to treat obesity are required.

Our results, together with previously published studies, highlight the need to consider region-specific analysis of the gut microbiota in order to fully understand the bacterial basis for the development of such diseases as obesity and the response to surgical and non-surgical treatment, opening to the possibility that probiotic development to treat different kinds of dysbiosis should be country-specific.

## Conclusion

This study identified significant differences in the human gut microbiota of obese patients from around the world, and found that functional dissimilarities were mediated by differences in taxonomic microbiota composition, which were region-specific, rather than alterations in metabolic pathways. This indicates the presence of functional metabolic redundancy between the microbiota of obese patients despite the bacterial differences and geographic origin. Furthermore, functional changes in gut microbiota following bariatric surgery were observed to be specific to the type of treatment received, providing new insights into the role of the gut microbiome in treatment strategies.

## Ethics Statement

All experiments were conducted in accordance with the Declaration of Helsinki and approved by the Ethics Committee of the Faculty of Medicine, Pontifical Catholic University of Chile (n° 15–337). DNA samples used here for metagenomic belongs from previous published studies (Medina et al., 2017). The 16S and metagenomic raw data sequences used belongs from previous published studies (Tremaroli et al., 2015; Yasir et al., 2015; Palleja et al., 2016; Ilhan et al., 2017; Medina et al., 2017; Thomson et al., 2019).

## Author Contributions

DM conceived, designed, performed the comparisons, analyzed the data, and wrote the manuscript. TL wrote and edited the figures and manuscript. PT contributed to the discussion. AA provided the scripts to do the multivariate data analysis. VP-B and AM critically read and edited the manuscript.

## Conflict of Interest

The authors declare that the research was conducted in the absence of any commercial or financial relationships that could be construed as a potential conflict of interest.

## Footnotes

1 http://huttenhower.sph.harvard.edu
