# Supplementary material for: Cross-Regional View of Functional and Taxonomic Microbiota Composition in Obesity and Post-obesity Treatment Shows Country Specific Microbial Contribution
Source: Front Microbiol. 2019 Oct 17;10:2346. doi: 10.3389/fmicb.2019.02346 (PMC6812679; doi:10.3389/fmicb.2019.02346)
Supplement: Supplementary file 6 [file Image_1.pdf]

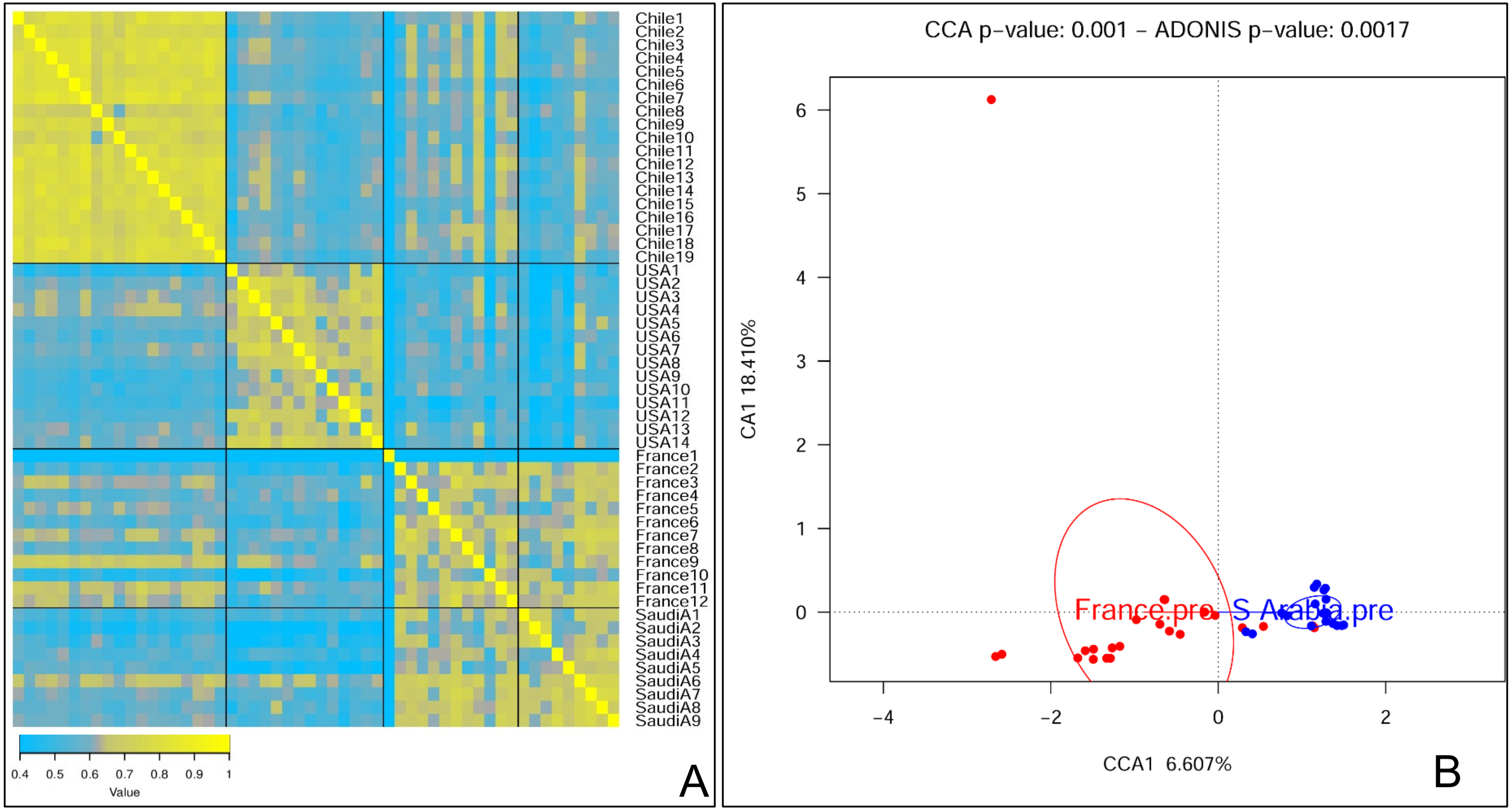

Supplementary Figure 1. Abundance correlation for gut microbiota and CCA composition comparison between France and Saudi Arabia. (A) Pairwise Spearman rank correlation for gut microbiota abundance between Chile, USA, France and Saudi Arabia subjects (from up to bottom and left to right respectively). (B) CCA and Adonis test comparing 16S rDNA sequence microbiota data obtained from obese patients from France (red) and Saudi Arabia (blue) at genus level.
