# Supplementary material for: Cross-Regional View of Functional and Taxonomic Microbiota Composition in Obesity and Post-obesity Treatment Shows Country Specific Microbial Contribution
Source: Front Microbiol. 2019 Oct 17;10:2346. doi: 10.3389/fmicb.2019.02346 (PMC6812679; doi:10.3389/fmicb.2019.02346)
Supplement: Supplementary file 7 [file Image_2.pdf]

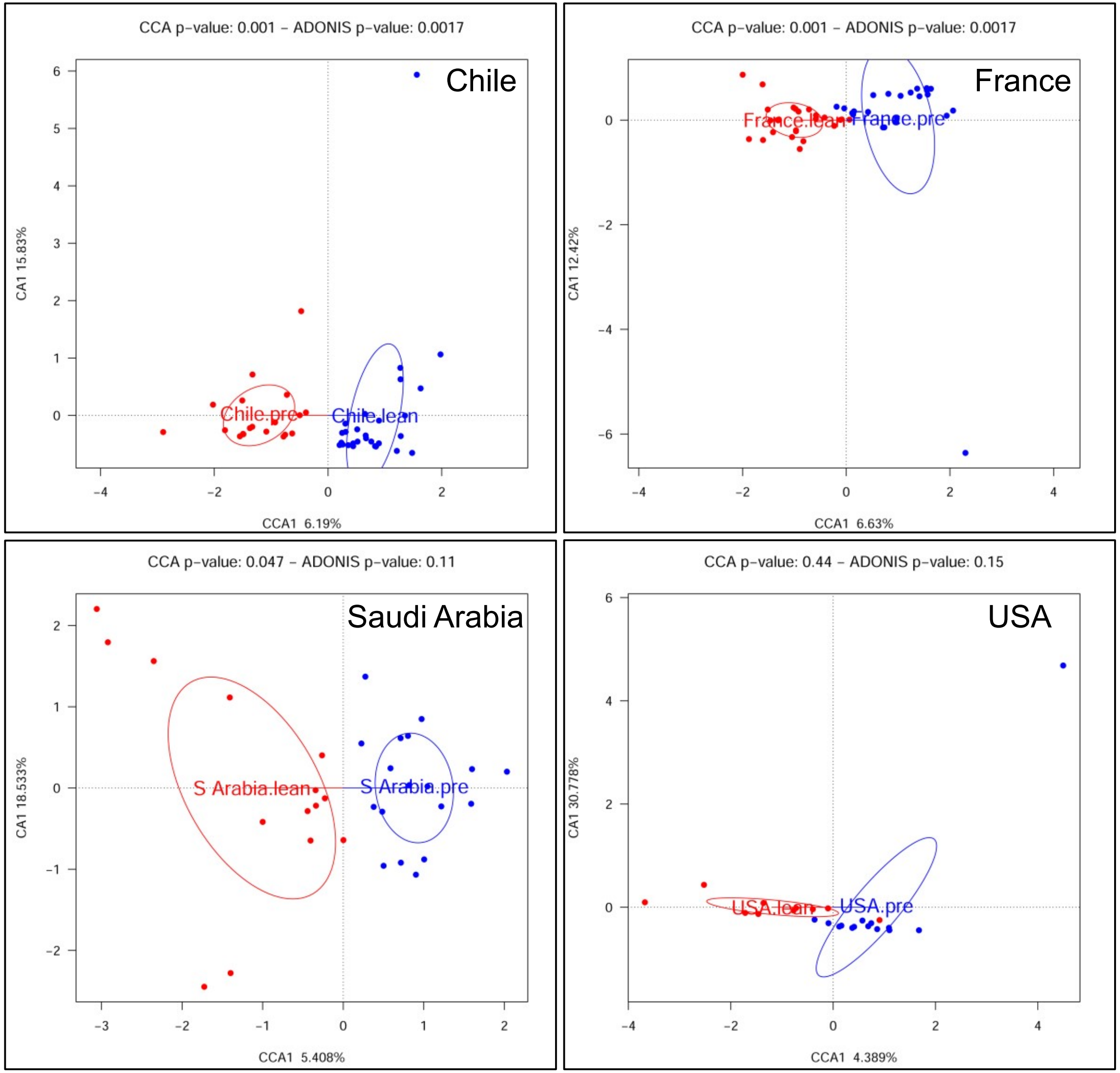

Supplementary Figure 2. Variance comparison between taxonomic abundances in lean and obese subjects. Constrained Correspondence Analysis (CCA) and Adonis test to contrast obese (red) and lean (blue) subject taxonomic abundance by country. p-values obtained from CCA and Adonis tests are denoted.
