# Supplementary material for: Cross-Regional View of Functional and Taxonomic Microbiota Composition in Obesity and Post-obesity Treatment Shows Country Specific Microbial Contribution
Source: Front Microbiol. 2019 Oct 17;10:2346. doi: 10.3389/fmicb.2019.02346 (PMC6812679; doi:10.3389/fmicb.2019.02346)
Supplement: Supplementary file 8 [file Image_3.pdf]

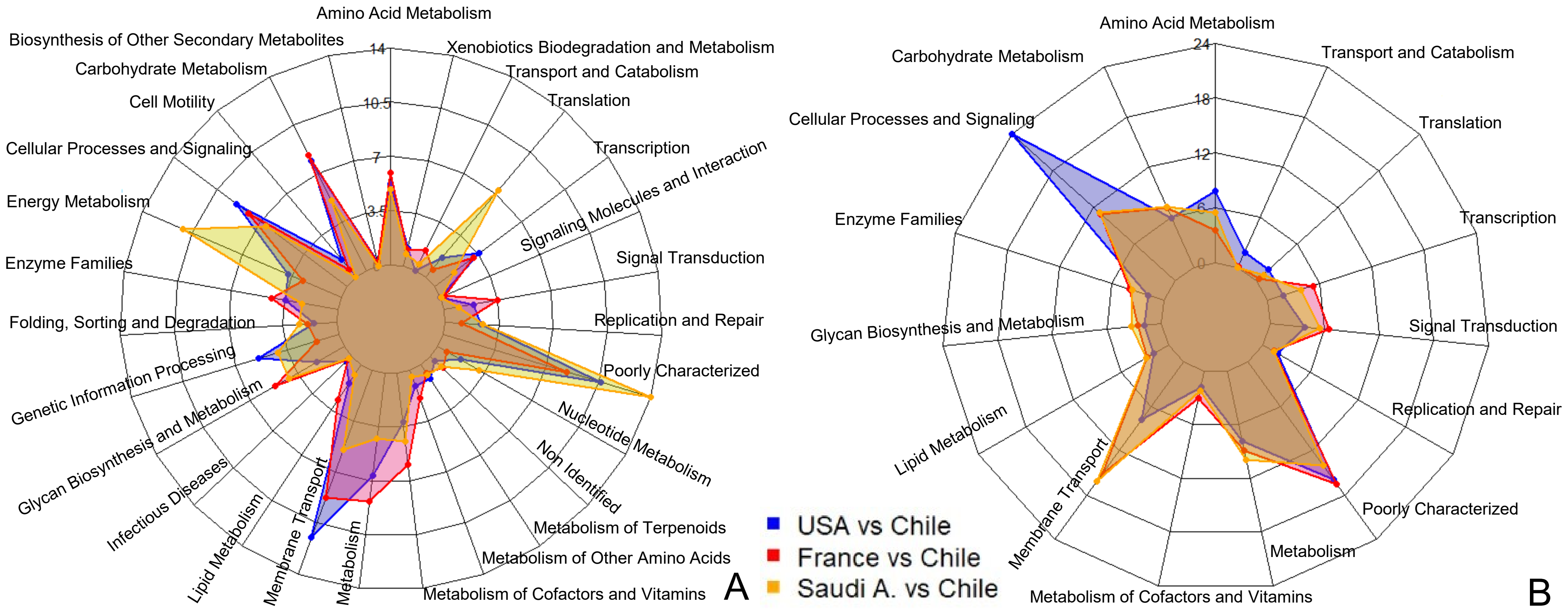

Supplementary Figure 3. Metagenomic assembled pathways from Kegg Orthology simulated data. Spider plot showing relative abundance of significantly downregulated (A) and upregulated (B) metabolic pathways shared between USA (blue), France (red) and Saudi Arabia (yellow) contrasted with Chile. Statistical differences were considered as FDR-adjusted p-value below of 0.05.
