# Supplementary material for: Cross-Regional View of Functional and Taxonomic Microbiota Composition in Obesity and Post-obesity Treatment Shows Country Specific Microbial Contribution
Source: Front Microbiol. 2019 Oct 17;10:2346. doi: 10.3389/fmicb.2019.02346 (PMC6812679; doi:10.3389/fmicb.2019.02346)
Supplement: Supplementary file 9 [file Image_4.pdf]

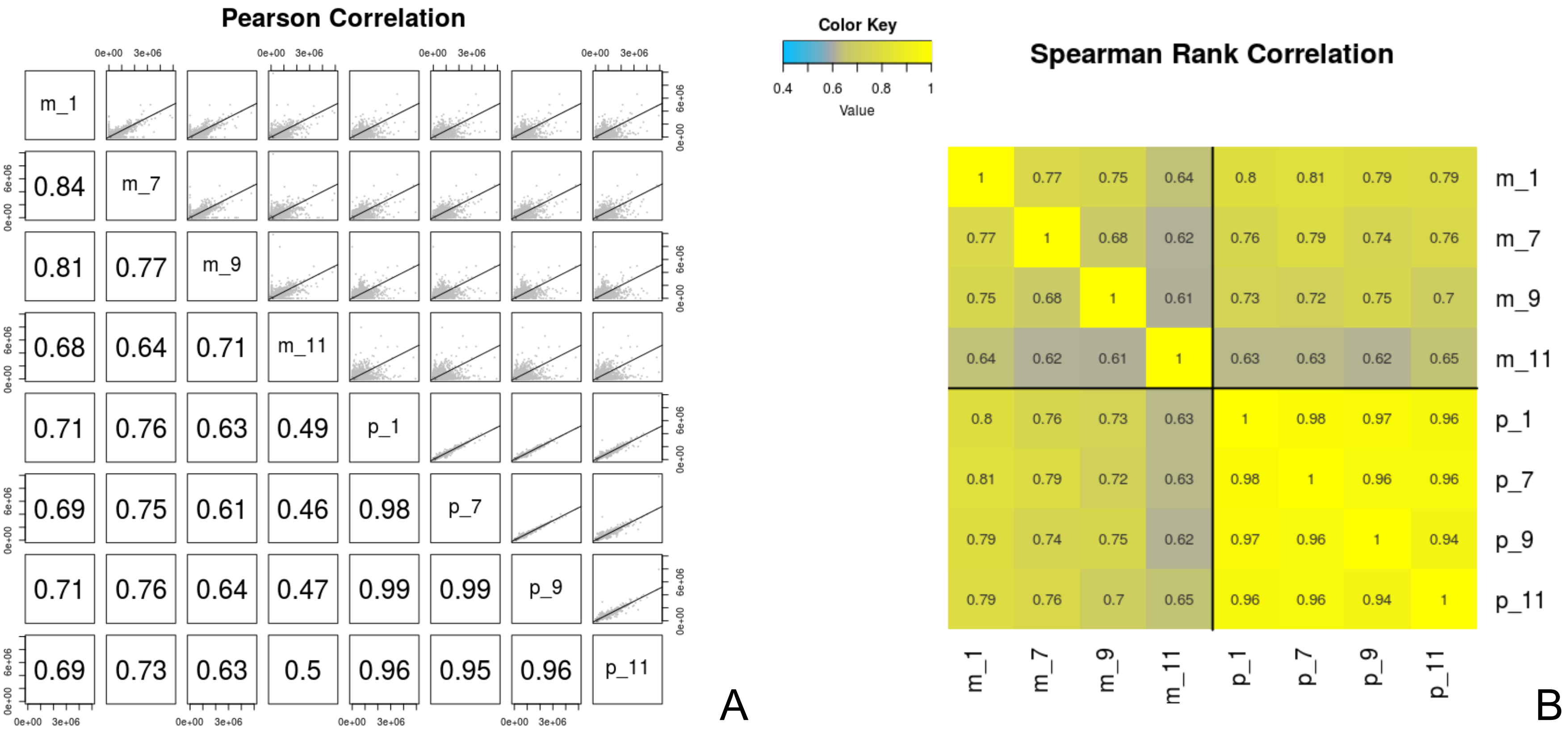

Supplementary Figure 4. Functional Prediction Validation. Pearson correlation (A) and pairwise Spearman Rank correlation (B) between KEGG Orthology metagenomic data (denoted by m_) and functional KEGG Orthology prediction by PICRUSt (denoted by p_). Numbers 1, 7, 9 and 11 represent subject codes.
