# Supplementary material for: Cross-Regional View of Functional and Taxonomic Microbiota Composition in Obesity and Post-obesity Treatment Shows Country Specific Microbial Contribution
Source: Front Microbiol. 2019 Oct 17;10:2346. doi: 10.3389/fmicb.2019.02346 (PMC6812679; doi:10.3389/fmicb.2019.02346)
Supplement: Supplementary file 10 [file Image_5.pdf]

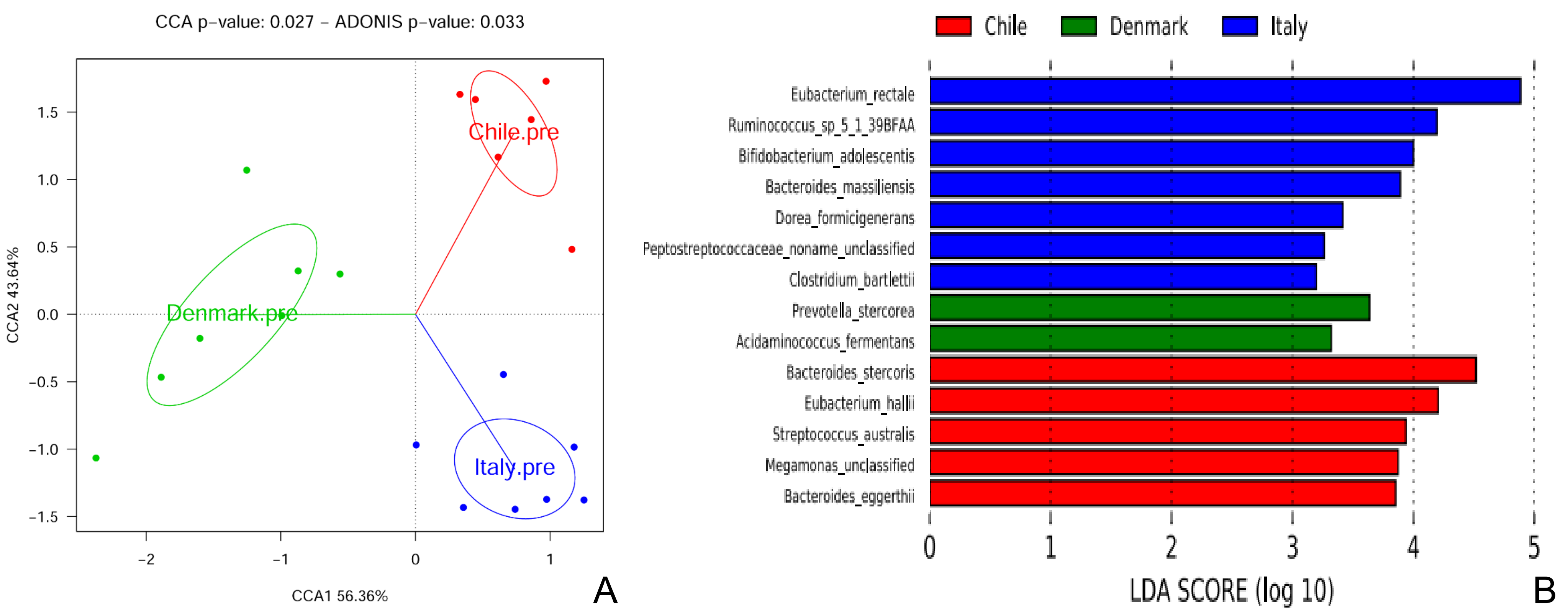

Supplementary Figure 5. Taxonomic abundance comparison at species level for Shotgun DNA sequencing data. (A) CCA and Adonis test comparing microbiota composition between Chile (red), Denmark (green) and Italy (blue). (B) Linear discriminant analysis (LDA) effect size (LEfSe) of enriched species with LDA scores higher than 2.
